# Patient and family experience with chronic transfusion therapy for sickle cell disease: A qualitative study

**DOI:** 10.1186/s12887-020-02078-w

**Published:** 2020-04-18

**Authors:** Lauren M. Hawkins, Cynthia B. Sinha, Diana Ross, Marianne E. M. Yee, Maa-Ohui Quarmyne, Lakshmanan Krishnamurti, Nitya Bakshi

**Affiliations:** 1 Emory University School of Medicine, Atlanta, GA USA; 2 Division of Pediatric Hematology-Oncology-BMT, Emory University School of Medicine, Atlanta, GA USA; 3 Aflac Cancer and Blood Disorders, Children’s Healthcare of Atlanta, Atlanta, GA USA

**Keywords:** Sickle cell, Chronic transfusion, Blood transfusion, Quality of life, Qualitative

## Abstract

**Background:**

There is a limited understanding of the patient and family experience of Chronic Transfusion Therapy (CTT) for prevention of complications of Sickle Cell Disease (SCD). We sought to understand patient and family experience with CTT using qualitative methods.

**Methods:**

Fifteen parents of children < 18 years old and nine children 12–18 years old with SCD who were receiving CTT for > 1 year were interviewed using a semi-structured interview format, and interviews were analyzed using open coding methods.

**Results:**

Four themes created a narrative of the patient and family experience of CTT: 1) Burden of CTT, 2) Coping with CTT, 3) Perceived benefits and risks of CTT, and 4) Decision making regarding CTT. Participants reported substantial burden of CTT, including the impact of CTT on daily life and family, distress about venous access, burden of chelation therapy, and anxiety about CTT complications. Participants described how they coped with CTT. Participants reported increased energy, decreased pain, fewer hospitalizations, and stroke prevention with CTT, but also recognized complications of CTT, though awareness was limited in adolescents. Parents described sharing in the informed decision-making process with their healthcare provider about CTT, but adolescent patient participants reported that they were not involved in this process.

**Conclusions:**

CTT is associated with significant patient and family burden. Support from family, healthcare providers and school may help individuals cope with some of this burden. These findings provide the basis for future studies to identify strategies to mitigate the burden of CTT and improve the patient experience with this therapy. Future studies should also systematically assess patient knowledge about the key components of CTT and chelation using quantitative assessments.

## Background

Chronic transfusion therapy (CTT) reduces or prevents sickle cell disease (SCD)-related complications. Specifically, CTT is very effective for the primary and secondary prevention of stroke [1–3], prevention of recurrent silent cerebral infarcts [4], and reduction of recurrent acute chest syndrome events [5] and hospitalization for pain [6]. However, CTT is also associated with complications such as transfusional iron overload [7], red cell alloimmunization [8], transfusion reactions, and the potential for infectious disease transmission [9, 10].

Parents of children with SCD receiving CTT report improved Health-Related Quality of Life (HR-QOL) [11] with better physical functioning, less bodily pain, and improved overall health when compared to parents of children not on CTT [11]. While survey-based methods measure HR-QOL and allow for quantitative comparisons, they provide a limited understanding of the entirety of the patient and family experience of CTT in SCD. Understanding patient and family experience is a key step in moving towards patient-centered care [12], and may serve as a starting point to identify strategies to improve patient experience. Good patient experience, which is an important outcome in itself, is also associated with improved clinical outcomes [13]. Qualitative research methods [14] allow for a rich, in-depth description of the patient experience, and can thus fill the knowledge gap in understanding patient and family experience.

In this study, we used qualitative interviews with parents and adolescent patients to understand the patient and family experience of CTT, the impact of CTT on their lives, their understanding of risks and benefits of CTT, and the decision making process surrounding CTT.

## Methods

English-speaking parents of children with SCD < 18 years of age receiving CTT (Group 1) and adolescents age 12–18 receiving CTT (Group 2) who were followed at a large comprehensive SCD clinic and had received CTT for more than a year were included in this study. Types of transfusions included simple, partial manual exchange, or automated erythracytapheresis. Children and parents of children who were weaning from CTT to hydroxyurea (HU) were included if they were receiving transfusions at the time of the study. There were no specific exclusion criteria.

We used a semi-structured interview guide (Additional file 1) with probes and follow-up questions determined based on the responses of the interviewee. An iterative process was used to make minor edits to the interview guide based on themes that emerged during the interviews. One in-person interview was conducted per participant, and if both the patient and the parent participated, then each participant was interviewed separately. A trained interviewer (LMH) conducted all of the interviews. The interviewer LMH received training in qualitative methods prior to start of the study, which included didactic instruction and role-play practice followed by feedback. This training was done over multiple sessions with study authors CS, DR and NB who were previously trained in qualitative methods. At the start of the study, some interviews included NB to ensure quality control. Interviews lasted 15–60 min, and were audio recorded and transcribed for analysis. We measured SCD-specific HR-QOL using the age-appropriate child or parent-proxy versions of PedsQL™ (PedsQL™, copyright© 1998 JW Varni, PhD, all rights reserved) Sickle Cell Disease Module [15, 16], which has evidence of validity and reliability [15, 16]. Scores range from 0 to 100 and higher scores indicate better HR-QOL. We collected demographic information from all participants and reviewed medical records for each child with SCD.

Demographic and clinical characteristics were analyzed using descriptive statistics using Stata Version 13. For qualitative interviews, the interviewer started analysis with line-by-line open coding as described by Corbin and Strauss [14]. Open coding is the ‘analytic process by which concepts are identified and their properties and dimensions are discovered in data’ [14]. Categories of 1) Experience of CTT, 2) Perceived risks and benefits of CTT and 3) Decision making about CTT were identified a priori based on literature review. Once we began coding, we developed additional significant categories that were central to the CTT experience. We based these additional categories on patterns that emerged in the coding scheme, and then merged these categories into over-arching themes. These themes together created a broad narrative understanding of family and patient experience with, and perception of CTT as a treatment option for SCD. We organized coding using NVivo Software Version 11. Two investigators (LMH and NB) developed the coding scheme, and a third investigator (CBS) substantiated the coding scheme.

All study procedures were approved by the Emory University Institutional Review Board. Written informed consent was obtained from all study participants. Written informed consent was obtained from parents if participants were < 18 years of age, and assent was obtained from the child.

## Results

### Demographic and clinical characteristics

We approached a convenience sample of 37 patient/parent dyads between June–December, 2018. Of the parents approached, seven parents of children < 12 years old, and eight parents of children ≥12 years old, participated in this study (Group 1). Of the adolescent patients approached, nine patients ≥12 years old participated in this study (Group 2), including six patients whose parents also participated. In total, we interviewed 24 participants, representing the experience of 18 unique patients. Demographic characteristics and HR-QOL of Group 1 and 2 participants (Tables 1 and 2, respectively), and clinical characteristics of the 18 unique patients represented in this study (Table 3) are described.

**Table 1:** Parent characteristics. Demographic characteristics for parent participants: age, gender, race, ethnicity, education level, employment status, income, and marital status

Parent Participants, Group 1 (*n* = 15)	
Age, Median (IQR)	41 (37–46)
Female gender, n (%)	13 (86.7)
African American Race, n (%)	13 (86.7)
Non-Hispanic Ethnicity, n (%)	14 (93.3)
Highest Education Level, n (%)	
High School Graduate or GED	2 (13.3)
Some College	3 (20)
Trade School/ Associate	2 (13.3)
Bachelors’ Degree	4 (26.7)
Masters’ Degree	4 (26.7)
Employment Status, n (%)	
Full time employment	11 (73.3)
No employment	3 (20)
Not Answered	1 (6.7)
Income, n (%)	
< $20,000/year	2 (13.3)
$20,000–$40,000/year	1 (6.7)
$40,000–$60,000/year	3 (20)
> $60,000/year	6 (40)
Not Answered	3 (20)
Marital Status, n (%)	
Single	4 (26.7)
Married	7 (46.7)
Separated or Divorced	4 (26.7)

**Table 2:** Patient Characteristics: Demographic characteristics for : Children of parent participants in Group 1 (excluding those patients represented in Group 2), and Group 2 (adolescent patient participants)

	Children of parents in Group 1 (*n* = 9)^a^	Group 2 (*n* = 9)
Age, Median (IQR)	10 (6–11)	14 (14–16)
Female gender n (%)	6 (66.7)	5 (55.6)
African American Race, n (%)	7 (77.8)	9 (100.0)
Non-Hispanic Ethnicity, n (%)	6 (66.7)	9 (100.0)
Education Level, n (%)		
Not yet in school	2 (22.2)	0
In School	7 (77.8)	8 (88.9)
High School Graduate	0	1 (11.1)
Patient HRQOL Score by self-report (*n* = 9), median (IQR)	–	90.7 (74.4–93)
Parent-proxy HRQOL Score, median (IQR)		
All parents (*n* = 15)^b^	68 (57.6–73.8)	
Parents not part of dyad (*n* = 9)	63.7 (57.6–73.8)	

^a^Does not include those patients represented in Group 2

^b^Includes parent-proxy HRQOL scores of some patients included in Group 2

**Table 3:** Clinical Characteristics. SCD-related clinical characteristics for patients: Children of parent participants (Group 1) and adolescent patient participants (Group 2) patients are both included here. Patients who were a part of a dyad are only represented once

Patient Characteristics (*n* = 18)	
Genotype, n (%)	
HbSS or HbS-β^0^ thalassemia	18 (100)
Transfusion Type, n (%)	
Simple	11 (61.1)
Erythracytapheresis	5 (27.8)
Partial manual exchange	2 (11.1)
Duration of CTT, median years (IQR)	6.8 (4.3–9.8)
Indication for CTT, n (%)	
Abnormal TCD	5 (27.8)
Overt or silent stroke	12 (66.6)
Splenic sequestration	1 (5.6)
Chelation type, n (%)	
None	3 (16.7)
Deferasirox	15 (83.3)
Taking hydroxyurea, n (%)	3 (16.7)
Current or prior CTT-related complications, n (%)	
Iron overload	17 (94.4)
Red cell antibodies	9 (50.0)
Port complications	3 (16.7)
Renal complications	2 (11.1)

### Qualitative analysis

Four major themes from the interviews created a narrative understanding of patient and family experience with CTT. These include: 1) Burden of CTT, 2) Coping with CTT, 3) Perceived benefits and risks of CTT, and 4) Decision-making regarding CTT.

#### Theme 1: Burden of CTT

Participants described many aspects of the burdens of receiving CTT (Table 4). The burden of care included the day-to-day logistical challenges of receiving CTT, including coordination and balancing of work, school, and family, challenges with venous access, burden of chelation therapy, and the emotional demands and worries associated with CTT.

**Table 4:** Burden of CTT

Categories	Salient quotations
Impact on daily life and family Work: Families report increased planning needs around work and challenges with having to miss work. Several describe challenges and increased demands of coordinating their schedules, and some report switching to night shifts to accommodate appointments, or having had to stop working. School: Patient and parents reported missing school, and distress associated with missing school, schoolwork, tests and school events, and having to make up schoolwork. Some adolescents reported feeling ‘frustrated’ or, ‘mad’ about missing school. A few parents reported concerns about support from school. Family: Parents report challenges with care for other children and family members, and increased planning/care-coordination needs. Transportation: Parents and patients report challenges with long commute times, traffic, parking, and difficulties with transportation for families without a personal vehicle	*“Because like I said I’m a single parent. So I have to work, so there’s not a lot of lee-way sometimes. Now we’re down to six employees and I had to take off today. I was so scared to call my supervisor today because, my God, I had to.”* (Parent) *“So he’s out Friday and today, which means he’s behind on somethings. He’s already struggling to do what he’s already doing. And this puts us even farther behind and a little more challenging. Is our biggest challenge with him, is being behind.”* (Parent) *“It’s kind of tough, I guess, if you’re learning. I mean it depends, cause if the teacher taught something new the day you’re out it’s kind of difficult to pick up on.”* (Patient)
Pain and distress with venous access Most patients and parents report pain, emotional distress, or fear associated with obtaining venous access. Some also report difficulty with obtaining venous access, need for central venous access and complications due to central venous access, and two adolescents commented on physical appearance of port.	*“And it’s kind of hard seeing your child, your little child in so much pain, you know, getting stuck. You know a lot of the times when they’re small they can’t find the veins. You know, I’ve watched them stick over and over and over again, you know, veins collapse. It’s, it’s just really a test. It really is. It’s a lot to go through.”* (Parent) *“Uh when I was younger they had people strap me down because I didn’t like needles, and I was terrified. And plus I couldn’t bear the pain.”* (Patient)
Emotional distress and worry surrounding CTT Parent participants expressed feelings of emotional distress surrounding their child receiving CTT, including feelings of stress and worry. Many parent participants specifically have uncertainty and worry about possible complications of CTT including iron overload, chelation, infection, organ damage, and unpredictable future events. Patient participants did not describe feelings of concern about potential future effects of transfusions.	*“It’s been a tough journey, its just a lot. For him to go through, for me to see him go through”* (Parent) “Um, I don’t know, cause we still like, we haven’t really figured that out, like how that’s gonna affect her body. So hopefully, Dr. *** said they can do an MRI of her liver or whatever. But um, I’m hoping for the best. That’s something that I’m fearful, very fearful about right now because I don’t want anything else like wrong with her. You know, especially something like that. So um, I’m hoping that’s gonna be okay. Praying.” (Parent)
Burden of Chelation The majority of patient and parent participants report challenges with taking chelation medications. Some of the challenges described include unpleasant taste of the powder forms of chelation medications, difficulty with remembering to take medications, distress with having to take multiple medications, and side effects.	*“He doesn’t like to look at them. Like he has the pill case. He covers it up on his dresser, and that bothers me for some reason because you have to take it. I don’t know why you don’t want to see it. He says he doesn’t want to see it.”* (Parent) *“And I hated it so much. Cause like, you have to mix it in your drinks and drink. And just does not feel good going down.”* (Patient)

#### Theme 2: Coping with CTT

Coping with the demands of CTT emerged as a key theme across interviews with the participants. Participants discussed how support from family, healthcare providers and staff, and schools were crucial in enabling them to cope with CTT.

Parents discussed how family members, including siblings, served as a support system for the patient receiving CTT including supporting the patient during hospital visits or at home, bringing the patient to appointments, and providing emotional support. One parent remarked,

*“And like I said, they* [her siblings] *see her when she come here and get the stick and get the blood, and, you know. So they don’t understand that part, but they’re very supportive. They make sure she’s fine. They know how to take care of her when she’s with them. They’re very watchful, mindful.”*

Most parents described a positive experience with healthcare providers and staff when they came for CTT appointments. A patient described how the positive clinic experience helped him not feel scared: *“Yeah, it’s like terrifying at first, but then once you start getting to know people or doctors you start feeling a little bit okay. Or sometimes you might want to suggest a doctor that you know for a long time so you don’t feel scared.”* A few patients and parents noted that forming close relationships with healthcare providers helped improve their experience with CTT. An adolescent participant explained that the head transfusion nurse, “*is like my hospital mom*,” which she noted made her feel more comfortable when coming to clinic. Another parent said, *“When we come, that helps us out a whole lot, how pleasant they are, how welcoming they are. They work really, really hard for us. You establish that relationship, so it’s kind of like coming home, away from home. So we’re not strangers in here by no means...”*

Many parents also highlighted the importance of support from their child’s school and teachers with schoolwork, accommodating absences, and catching up on missed schoolwork. One parent described how communication with the child’s teachers helped to make sure she had good support in place: “So I did a lot of education with them, so um, just so they could be mindful of how her eyes look, how her mood is. They could tell like, okay, it’s time for her to go get another transfusion ‘cause she’s starting to drag. So I educate them on a lot of stuff, and we was always in communications. They tell me, ‘Okay, she didn’t have a good day today. You may want to come on and get her.’ You know, so, we were always in communication … so last, ** grade year was great for her.”

Both patient and parent interviews also revealed that they became accustomed to the experience of CTT over time. Patients described this as “*get used to it*” or “*deal with it*,” or *“it’s kind of normal now”* for explaining how they adapted to the experience of CTT. Parents used terms like *“used to”* and *“it’s our way of life”*. One parent exemplified this sentiment: “*So after so long, and she’s so resilient, it kind of just, it was just like a normal routine. She’s gotten used to the sticks. She’s gotten used to the labs. So, yeah, … it’s just has become part of life. Like, we just accustomed to it. It’s the norm, this is our norm*.” Another parent said, *“it’s kind of, it’s almost robotic for us”*, in describing the days they come for CTT. Later on in the interview, the same parent remarked that her son’s long duration of CTT helped him to see it as a typical experience: *“I think he’s used to it only because he’s never known anything else. Um, probably until he was about ten years old he was just under the impression that every child did this. So he didn’t realize that he’s different. Um, so I think he’s comfortable with it because he doesn’t know any other life”.* Some parents also talked about how their child had *“accepted the process” or “come to terms with it”.* While parents described CTT becoming a routine experience over time, some of them still alluded to the challenges of CTT, and how they how they just had to *“make it work”* or *“I have to do what I have to do to make sure he’s OK”.* One parent said, *‘it was tough to accept at first, but this is something we have to do”.*

#### Theme 3: perceived benefits and risks of CTT

We asked participants to discuss their understanding of the transfusion process, including the benefits and potential risks of CTT. Participants described benefits and risks during the course of the interview and in response to open-ended questions, and in many (but not all) instances, the interviewer probed for awareness of some of the specific benefits and risks if they were not brought up by the participant.

Almost all parents and majority of patient participants were aware of stroke prevention as a benefit of CTT. Both patients and parents also described reduction in painful events with CTT, though parents were more explicit in their description of reduction in pain events. The majority of parents also reported improvement in their child’s energy levels following a transfusion, and some talked about decrease in other sickle cell complications or hospitalizations.

Almost all parents and majority of patients were aware of the risk of iron overload with CTT. Only a few parents were aware of risks of alloimmunization or antibody development, but adolescent patients did not express awareness of this risk at all. Many parents had concerns about risk of infection associated with transfusions. Parents overall appeared to have more awareness of risks as compared to adolescents. In some instances, participants reported first learning about risks or complications after CTT had started or when they had experienced them.

We specifically elicited participant understanding of why they were receiving CTT, and corroborated responses with the stated indication for CTT in the medical record. All parents identified the correct indication for their child, while only five of nine adolescent patient participants described the correct indication.

#### Theme 4: decision making about CTT

Almost all parents felt involved in the decision-making process when CTT was initiated for their child. These parents recalled having conversations with the physician about CTT. However, some parents specifically highlighted that their decision to pursue CTT was presented as the only reasonable option. This was emphasized in a parent’s description of the need for transfusions: *“Um, really wasn’t that big of a decision. It was either blood transfusion or he will have another stroke. So um, and as you see, even with the blood transfusions, there still is a risk of him having another stroke. But, it wasn’t, I didn’t have a choice.”*

Adolescent participants reported not being involved in the decision making process for CTT, but all of them had started on CTT at ≤12 years of age. One patient described that he remembered the start of CTT, but he did not recall having the transfusions explained to him as they began*, “I dunno how it happened, ‘cause I used to just come in for regular check-ups. And then I guess, it just happened, that I got started getting blood transfusions.”*

The interviewer also asked participants if they wished they had known more about CTT, especially when they started CTT. Some parents reported that they would have wanted to know more about the risks of CTT, such as iron overload:

*“Going in, I didn’t know, I didn’t know that the iron that’s in the blood would cause her to have extra iron. I didn’t know that our bodies cannot release the iron naturally, that we have to have a medication. I didn’t know that that would be an issue when she first started getting transfusions. I might have looked into it a little more carefully and I might have looked to see if there was another option. Um, where she is right now, her levels are so high that to kind of stop now would just be ridiculous. So, yeah, had I been more informed about certain things I probably would have opted for her not to get them.”*

## Discussion

This in-depth qualitative study comprehensively describes the multiple dimensions of the patient and family experience related to receiving CTT in SCD, which have not been previously reported. This study is complementary to, and extends the findings from previous survey based studies of HRQOL in children receiving CTT for SCD [11, 17]. The findings of this qualitative study are also consistent with a prior qualitative study by Stegenga *et. al*, who interviewed 10 pediatric patients to describe the impact of CTT on school and identified perceived benefits of CTT as stroke prevention and improved energy [18].

We found that patients and families experience substantial burdens of care and challenges in balancing demands of work, school, and other life activities with CTT, though they recognize the importance and benefits of CTT. This is similar to the perspective of parents of children with other chronic illnesses, who report challenges of balancing work and family, time constraints, and stress associated with their child’s care [19] and have decreased odds of employment [19, 20]. It is possible that the significant burden of care contributes to difficulties with adherence to CTT, which is sub-optimal even among children enrolled in clinical trials of SCD. In the SWITCH study, 34% of patients had ≥1 late transfusion (defined as transfusion outside 7-day window of scheduled visit), and 12% of patients had ≥2 late transfusions prior to entering the study [21]. While healthcare providers are cognizant of the burdens [22], this study provides a detailed description from the perspective of patients and their parents of how their lives are impacted by CTT. These findings can provide guidance in the design and implementation of systems to minimize the burdens placed on patients and their caregivers.

Children with SCD experience more problems with academic attainment as compared to their peers [23]. School absenteeism was a concern for both patients and parents in this study, and may be of importance because illness-related school absences predict academic attainment [23] in some studies. Children with both overt and silent strokes also have impaired neuropsychological function [24], and school performance may be further impacted adversely impacted by the neurological complications of SCD, such as silent infarcts [25]. Patients receiving CTT may thus face academic difficulties not only from SCD related neurological and neurocognitive dysfunction, but also from school absences due to receiving CTT. Adolescents in this study also expressed distress with having to ‘catch-up’ and miss other school related activities, similar to previous work by Stegenga et al. [18]. Healthcare providers should recognize these barriers and consider measures to minimize school disruption due to CTT.

Families also experienced burden through the challenges they had with chelation. To our knowledge, this is the first qualitative study to describe challenges with oral chelation therapy in pediatric patients with SCD. Even though this is a small cohort of pediatric patients with SCD on CTT, participants highlighted the burden of daily chelation therapy. Previous work in children with thalassemia major who received desferrioxamine has highlighted how adolescents perceived chelation therapy as disruptive, how it was central to their illness narrative, and how it marked out their ‘difference’ when compared to their peers [26]. The perceived burden and distress surrounding chelation observed in our study, suggests that healthcare providers should specifically address challenges with chelation at outpatient transfusion visits. Tools to assess patient satisfaction with iron chelation therapy may be helpful to guide providers in therapy management [27].

Participants identified several methods of coping with the stressors of CTT, especially through support systems. Sources of support, both within medical systems and outside of them, appear to be valuable to the patient experience of CTT. Previous studies have described the importance of social support in the management of chronic illnesses such as diabetes [28], and this may also positively influence the outcomes of chronic illness, regardless of self-management behaviors [28]. Some parents in this study noted that peer support may also have been helpful. Strategies to mitigate burdens by expanding options for parent and child support may be useful, and could also include peer support from other patients and families who have prior experience with CTT.

The concept of ‘normalization’ of the CTT experience emerged during interviews. Patients and parents described how they got accustomed to the process of CTT, and how it became part of their life. Some parents also described how the child’s getting “used to” CTT made the process easier. Robinson [29] describes that families construct a story of life ‘as normal’, which involves minimizing significance of problems with the chronic condition, enabling ‘looking at the bright side’, and by reconstruction of reference points by which the experience is judged. Deatrick et al. [30] have described the five attributes of ‘normalization’, which are 1) acknowledging the condition and potential threat to lifestyle, 2) adopting a ‘normalcy’ lens for child and family, 3) engaging in parenting behaviors and family routines consistent with the normalcy lens, 4) developing a treatment regimen consistent with normalcy, and 5) Interacting with others with view of child and parent as normal. Other elements include acceptance of the child’s condition and changing expectations for the child [31]. The participants in our study expressed several of these elements of normalization. This is similar to the experience in other pediatric chronic illnesses [31–33], where this concept of ‘normalization’ has been expressed.

The majority of parents in this study described an informed decision-making process with the child’s physician when initiating CTT, though several parents expressed that they understood CTT to be their only reasonable option. This is not surprising because physicians generally adopt a ‘proponent approach’ while discussing CTT, in which the physician advocates for initiating a particular treatment and convinces patients/families to adopt this treatment, without consideration of an alternative decision (i.e. not to initiate CTT) [22] because CTT is often used to prevent some of the severe complications of SCD, such as stroke. While we did not use a structured survey to assess knowledge of risks and benefits of CTT, to the extent that we were able to ascertain from interviews, adolescent patients with SCD had appeared to have limited understanding of risks of CTT, which has also been demonstrated in other studies [34]. This may have been in part due to the young age of initiation of CTT, and lack of involvement in decision making, but suggests that adolescents would likely benefit from education about CTT, even if they have received CTT for many years. These considerations may be especially important as adolescents on CTT transition to adult care.

Limitations of this study include the use of a convenience sampling strategy. The experiences described in this study reflect only those of parents and adolescents who chose to participate, and thus may represent individuals who overall may have had more positive experiences with CTT or were more adherent with CTT. Additionally, patients whose parents were unable to attend clinic visits in person were not included in this study. This group potentially has unique burdens and experiences related to CTT, which could not be captured. There may also have been social desirability bias in the participant responses. We conducted this study in a single hospital system, and thus the results of this study may not be generalizable to the larger patient population. The majority of participants were female, especially mothers, and their views may not be shared by fathers or other caregivers. The small sample size precluded the study of the role of demographic variables such as education, income, marital status and employment with the themes identified in the study. While our study identifies gaps in patient and family understanding of CTT, we did not formally assess knowledge using surveys, and are thus unable to make quantitative assessments regarding knowledge of CTT.

## Conclusions

CTT is associated with significant patient and family burden. Support from family, healthcare providers and school may help individuals cope with some of this burden. These findings provide the basis for future studies to identify strategies to mitigate the burden of CTT and improve the patient experience with this therapy. Future studies should also systematically assess patient knowledge about the key components of CTT and chelation using quantitative assessments.

## Supplementary information

**Additional file 1.** Semi-structured Interview Guide.

## Data Availability

Data are not available. The data consist of individual interview transcripts, which cannot be made publicly available due to privacy concerns.

## Abbreviations

CTT: Chronic transfusion Therapy
SCD: Sickle Cell Disease
HR-QOL: Health Related Quality of Life
HU: Hydroxyurea
