# Supplementary material for: Patient and family experience with chronic transfusion therapy for sickle cell disease: A qualitative study
Source: BMC Pediatr. 2020 Apr 18;20:172. doi: 10.1186/s12887-020-02078-w (PMC7165370; doi:10.1186/s12887-020-02078-w)
Supplement: Supplementary file 1 — Additional file 1. Semi-structured Interview Guide. [file 12887_2020_2078_MOESM1_ESM.docx]

**Additional file 1: Semi-structured Interview Guide**

Interviewer used this guide to structure order and content of interviews, but the exact wording, order or content of questions may have differed. Probes (in italics) served as a guide for discussion and follow-up of participant responses.

| 1. Tell me a bit about what happens on transfusion day.    1. What happens on a typical transfusion day?       1. *Timing; What you/child does during transfusions; Transportation, costs; Providers(s) seen; Interference with school/work/etc*    2. After the transfusion (day of CBT) how does your child feel?       1. *Medical/physical; Emotional; School performance* 2. I have a few questions about your overall transfusion experience.    1. What are some improvements you have seen in you/your child since starting chronic blood transfusions?       1. *Physical; Medical (what do the doctors say?); Social; Emotional*    2. What are some complications you have seen in your child since starting chronic blood transfusions?       1. *Physical; Medical (what do the doctors say?); Social; Emotional* 3. I would also like to understand more about you/your child’s decision to be treated with chronic transfusions.    1. How did you/your child make the decision to start chronic blood transfusions?       1. *What were they told SCD provider; Other sources of information; Their understanding of reason for starting therapy*    2. Who helped make the decision to start this therapy?       1. *Doctor/other healthcare provider; Parents; Others; Child’s role*    3. What was you/your child’s understanding of the benefits of this therapy before you started this therapy?    4. How has your understanding of benefits of chronic blood transfusion changed since starting therapy?       1. *Decrease in complications (stroke, ACS); Decrease in pain; Fewer hospitalizations*    5. What was you/your child’s understanding of the risks/complications before you started this therapy?    6. How has your understanding of risks/complications of chronic blood transfusion changed since starting therapy?       1. *Alloimmunization; Iron overload; Infectious risk*    7. What were some things that surprised you about this therapy that were different from your expectations?       1. *Benefits; complications; logistical challenges*    8. Is there something you wish you/your child would have known about this therapy before you started it?       1. *Benefits; complications; logistical challenges* 4. If you were talking to someone who was about to start getting chronic transfusions, what would you tell them? 5. Is there anything else about chronic blood transfusion that you wish to discuss with me? |
| --- |
